# Bioprocess Control in Microscale: Scalable Fermentations in Disposable and User-Friendly Microfluidic Systems

**DOI:** 10.1186/1475-2859-9-86

**Published:** 2010-11-13

**Authors:** Matthias Funke, Andreas Buchenauer, Wilfried Mokwa, Stefanie Kluge, Lea Hein, Carsten Müller, Frank Kensy, Jochen Büchs

**Affiliations:** 1 AVT-Biochemical Engineering, RWTH Aachen University, Worringerweg 1, D-52074 Aachen, Germany; 2 Institute of Materials in Electrical Engineering 1, RWTH Aachen University, Sommerfeldstr. 24, D-52074 Aachen, Germany; 3 m2p-labs GmbH, Forckenbeckstraße 6, D-52074 Aachen, Germany

## Abstract

**Background:**

The efficiency of biotechnological production processes depends on selecting the best performing microbial strain and the optimal cultivation conditions. Thus, many experiments have to be conducted, which conflicts with the demand to speed up drug development processes. Consequently, there is a great need for high-throughput devices that allow rapid and reliable bioprocess development. This need is addressed, for example, by the fiber-optic online-monitoring system BioLector which utilizes the wells of shaken microtiter plates (MTPs) as small-scale fermenters. To further improve the application of MTPs as microbioreactors, in this paper, the BioLector technology is combined with microfluidic bioprocess control in MTPs. To realize a user-friendly system for routine laboratory work, disposable microfluidic MTPs are utilized which are actuated by a user-friendly pneumatic hardware.

**Results:**

This novel microfermentation system was tested in pH-controlled batch as well as in fed-batch fermentations of *Escherichia coli*. The pH-value in the culture broth could be kept in a narrow dead band of 0.03 around the pH-setpoint, by pneumatically dosing ammonia solution and phosphoric acid to each culture well. Furthermore, fed-batch cultivations with linear and exponential feeding of 500 g/L glucose solution were conducted. Finally, the scale-up potential of the microscale fermentations was evaluated by comparing the obtained results to that of fully controlled fermentations in a 2 L laboratory-scale fermenter (working volume of 1 L). The scale-up was realized by keeping the volumetric mass transfer coefficient *k_L_a *constant at a value of 460 1/h. The same growth behavior of the *E. coli *cultures could be observed on both scales.

**Conclusion:**

In microfluidic MTPs, pH-controlled batch as well as fed-batch fermentations were successfully performed. The liquid dosing as well as the biomass growth kinetics of the process-controlled fermentations agreed well both in the microscale and laboratory scale. In conclusion, a user-friendly and disposable microfluidic system could be established which allows scaleable, fully controlled and fully monitored fermentations in working volumes below 1 milliliter.

## Background

State-of-the-art bioprocesses are based on a large number of small-scale experiments in which the best performing microbial strain and the optimal cultivation conditions are evaluated. These screening experiments are becoming even more important, since modern methods in genetic engineering and molecular biology can generate thousands of different clones. Moreover, biotechnological research - spurred e.g. by the process analytical technology (PAT) initiative of the FDA [1] - additionally identifies the influences of a growing number of media ingredients and process variables. All these clones and parameters are targets for screening and process optimization [2,3]. On the other hand, pressure is mounting on pharmaceutical companies, to cut costs and speed up the drug development processes [4]. Therefore, more microbial cultivations have to be performed in a shorter time. To solve this conflict, the need for high-throughput small-scale cultivation systems indeed increases. Such systems have to provide reliable data for process characterization and scale-up. Reliability, in this case, means to generate a high information output from small-scale cultivations conducted under well characterized conditions.

Many different miniaturized bioreactors have been developed to address the increasing demand on small-scale fermentation systems [reviewed in: [5-10]]. Among them are, for example, miniaturized bubble columns and stirred tank reactors. Yet in recent years, shaken microtiter plates (MTPs) as microbioreactors have also gained in importance [11,12]. Since decades, MTPs have been widely used in various applications in almost every biology laboratory. To place their application as shaken microbioreactors on a firm footing, intense efforts have been made to characterize fluid movement, gas transfer, energy input and mixing in these vessels [reviewed in: [11-13]]. This characterization work and the developed methods also build the fundament to improve the MTPs as described by Funke et al. [14]. The authors optimized the MTP-well geometry by introducing baffles and hereby could double the maximum oxygen transfer capacity (OTR_max_) and improve the online-monitoring in MTPs. This new MTP-well geometry, the Flowerplate (m2p-labs GmbH; Aachen, Germany), is also applied in this current work.

To further improve the application of MTPs as microbioreactors, active process control has been implemented. Buchenauer et al. [15] have described a microfluidic chip, replacing the bottom of a conventional MTP, which allows the transfer of nanoliter amounts of liquids from a reservoir well to a culture well in a controlled manner. The authors have applied this system in pH-controlled *Escherichia coli *cultivations. As described by Funke et al. [16], the concept of microfluidic process control in MTPs was further developed by integrating a micropump for controlled substrate feeding into the microfluidic chip. Thereby the authors have demonstrated the proof-of-principle for microfluidic driven pH-control and fed-batch cultivations in MTPs. After this proof-of-concept, in the current work this microfluidic process control system is verified by investigating the scale-up potential of the microfluidic bioprocess control. Moreover, here this concept is transferred to disposable microfluidic chips, which is the basis for routine-use, since the utilization of elaborate and costly dosing hardware such as syringes, pumps or robots can be avoided.

To transfer this technology to a more user-friendly and robust system, the layout of the microfluidic chip and its connection to the actuator hardware has subsequently been improved [17]. The pneumatic connections are integrated directly in the shaker tray and, thus, make it unnecessary to manually connect the microfluidic MTP to the tubes of the pneumatic actuator hardware. In this paper, this user-friendly connection of the microfluidic MTP to the pneumatic actuator hardware is applied in a ready-to-use microfermentation system.

In order to meaningfully apply MTPs as microbioreactors, an efficient online-monitoring with high information output is needed. The most promising approach to cope with this need is the technology first described by Samorski et al. [18] and meanwhile marketed as the BioLector technology by the m2p-labs GmbH (Aachen, Germany). This system allows the continuous detection of relevant fermentation parameters in conventional MTPs by utilizing fiber-optic, non-invasive measurements by means of optodes (pH and DOT), light scattering (biomass) or fluorescence (NADH, GFP etc.) [19]. This technique serves as the basis of the microfermentation system described here.

The current trend to process miniaturization is only meaningful, if the results obtained in small-scale fermentations are scalable to large-scale processes. The scale-up is successful provided that the cultivation conditions are comparable between the scales. However, it is not possible to keep all cultivation parameters constant upon converting the process to a larger scale. Thus, the parameter most important to the process has to be chosen as the scale-up criterion [20]. In most cases, the nature of the process and the properties of the microorganism dictate this choice [21]. If the oxygen transfer is known to be the limiting cultivation parameter, as it indeed is in most aerobic single-cell fermentations, scale-up is most often performed by keeping the oxygen transfer constant [22]. In *E. coli *cultivations, the concept of constant volumetric mass transfer coefficient *k_L_a *has been applied before for the scale-up of MTP fermentations to stirred tank reactors [23-25].

The microfermentation system described in this paper combines the four advantages mentioned above: (1) the improved MTP-design which allows higher oxygen transfer; (2) the disposable microfluidic chip for process control in MTPs; (3) the user-friendly hardware for connecting the microfluidic MTP to the pneumatic actuator hardware, and (4) the advanced online-monitoring with the BioLector technology. These advantages of the so-called 'microfluidic BioLector' concept allow one to perform scalable microbial cultivations on a microscale. Besides the test of this novel, user-friendly system in pH-controlled batch as well as in fed-batch fermentations of *Escherichia coli*, this paper describes the scale-up of the microfermentations and the comparison of the obtained results in the microfluidic MTP to fermentations in a 2 L laboratory-scale fermenter (working volume of 1 L).

## Materials and methods

### Microfluidic BioLector

**Figure 1:**
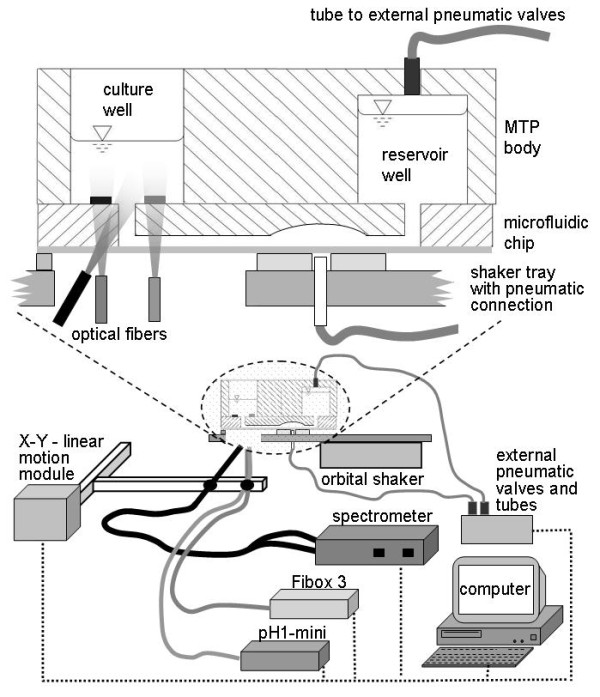
**Microfluidic BioLector**. The different parts of the BioLector measurement system is schematically shown in the lower part. The upper part of the figure depicts a magnification of the micro bioreactor setup with microfluidic control.

The microfluidic BioLector system consists of an adapted BioLector measurement system and the actuator hardware for the microfluidic MTP (Figure 1). The adapted system utilized in this work is composed of a modified orbital shaker (based on Lab-Shaker LS-W, Kühner AG, Basel, Switzerland), a x-y linear motion module (Bosch Rexroth AG, Lohr am Main, Germany), external pneumatic valves (MHP1; Festo, Esslingen, Germany) driven by a digital USB I/O device (National Instruments, Austin, TX, USA), a fluorescence spectrometer (Fluoromax 4P; HORIBA JobinYvon, Unterhaching, Germany) for optical biomass measurement as well as a pH1-mini and a Fibox 3 for optical pH and DOT measurement, respectively (both: PreSens GmbH, Regensburg, Germany). The light of each of these devices is directed to each individual well of a MTP by means of separate optical fibers. The optical fiber for scattered light measurement from the spectrometer is fixed at an angle of approx. 30° to one arm of the linear motion module in order to avoid interference by direct light reflection from the well bottom. The optical fibers of the pH1-mini and the Fibox 3 are mounted vertically, since the measurements of pH-value and DOT are based on the analysis of fluorescence lifetime and, thus, are not disturbed by light reflection. A LabVIEW program (National Instruments, Austin, TX, USA) controls the linear motion module in order to achieve a recurrent measurement in each individual well. Moreover, this program controls the external valves via the digital I/O device, and it acquires and finally saves the data from the spectrometer, the pH1-mini and the Fibox 3. The implemented orbital shaker has been modified by the company Kühner, to realize a shaking diameter of 3 mm and shaking frequencies of up to 1000 rpm. An interruption of oxygen supply and mixing during the measurement procedures is hereby avoided due to continuous shaking of the bioreactor. Moreover, to reduce evaporation, a hood is placed above the bioreactor setup on the shaker tray, and is flushed continuously with humidified air (not shown in Figure 1). The continuous shaking and the flushing with ambient air are sufficient to guarantee oxygen transfer rates of up to 100 mmol/L/h. An enrichment of the air flow with additional oxygen is not necessary.

**Figure 2:**
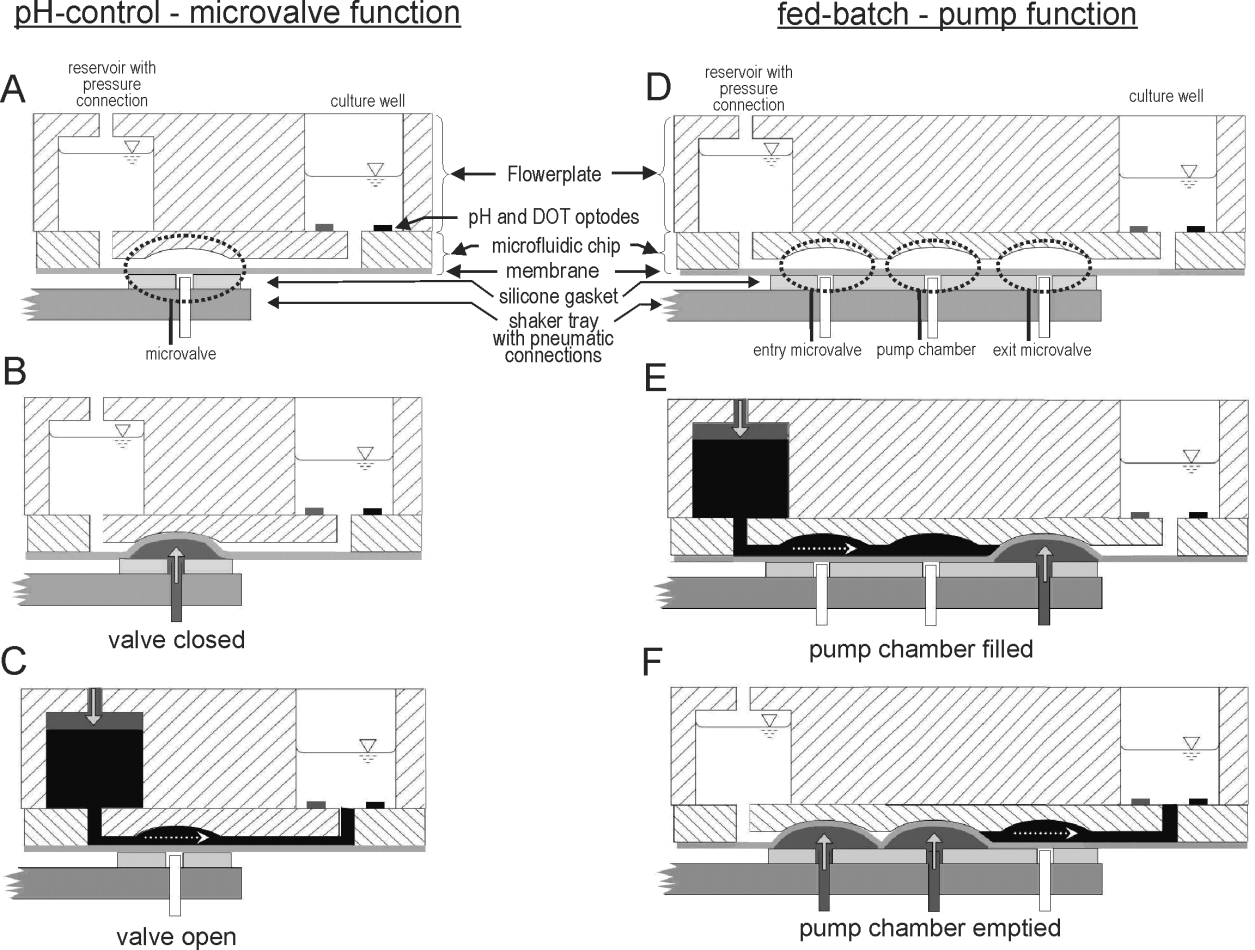
**Cross-section scheme and function of microfluidic MTP**. The two different working principles of the microfluidic process control are shown: the microvalve function applied in pH-control **(A-C) **and the micropump function, used for fed-batch fermentations **(D-F)**. Filled arrows and dark gray color indicate pneumatic pressure in the pneumatic channels and the reservoir wells. Dashed arrows and black color indicate moving liquid due to the pneumatic pressure.

The microfluidic MTP is mounted on the shaker tray of the BioLector orbital shaker. As Figures 1 and 2 illustrate, external valves control the flow of pressurized air via tubes to the pneumatic connections, which are integrated directly in the shaker tray. Moreover, the external valves direct pressurized air to the reservoir pressure connections on top of each reservoir well of the microfluidic MTP (Figure 1). (For detailed description see paragraph "Microfluidic MTP")

The measurement technique of the BioLector bases on the direction of light to the sample inside a MTP well by means of an optical fiber and concomitant detection of fluorescence and back-scattering [18,19]. For determining the biomass concentration, the back-scattered light is measured at an angle of 180° with the fluorescence spectrometer Fluoromax 4P (wavelength excitation and emission: 600 nm/slit-width: 5 nm). For each measuring point, the spectrometer software sums up the detector signals from 150 flashes (at 90 ms intervals) of the spectrometer's xenon flash lamp.

The measurements of the pH-value and the dissolved oxygen tension (DOT) are based on the lifetime measurement of an indicator which is sensitive to H^+^-ions and O_2_, respectively (optode). For pH-measurement, the fluorescence lifetime of a second indicator serves as a reference. This method, described by Huber et al. [26], is, therefore, called dual lifetime referencing. In addition, the measurement of DOT is based on dynamic luminescence quenching by molecular oxygen of the excited state of an indicator [27]. Since the fluorescence lifetime of the O_2_-sensitive indicator is longer and thus easier to measure, no reference indicator is needed. These principles for pH- and DOT-measurement are respectively applied in the utilized devices pH1-mini and Fibox 3 of the company PreSens (Regensburg, Germany).

### Microfluidic MTP

**Figure 3:**
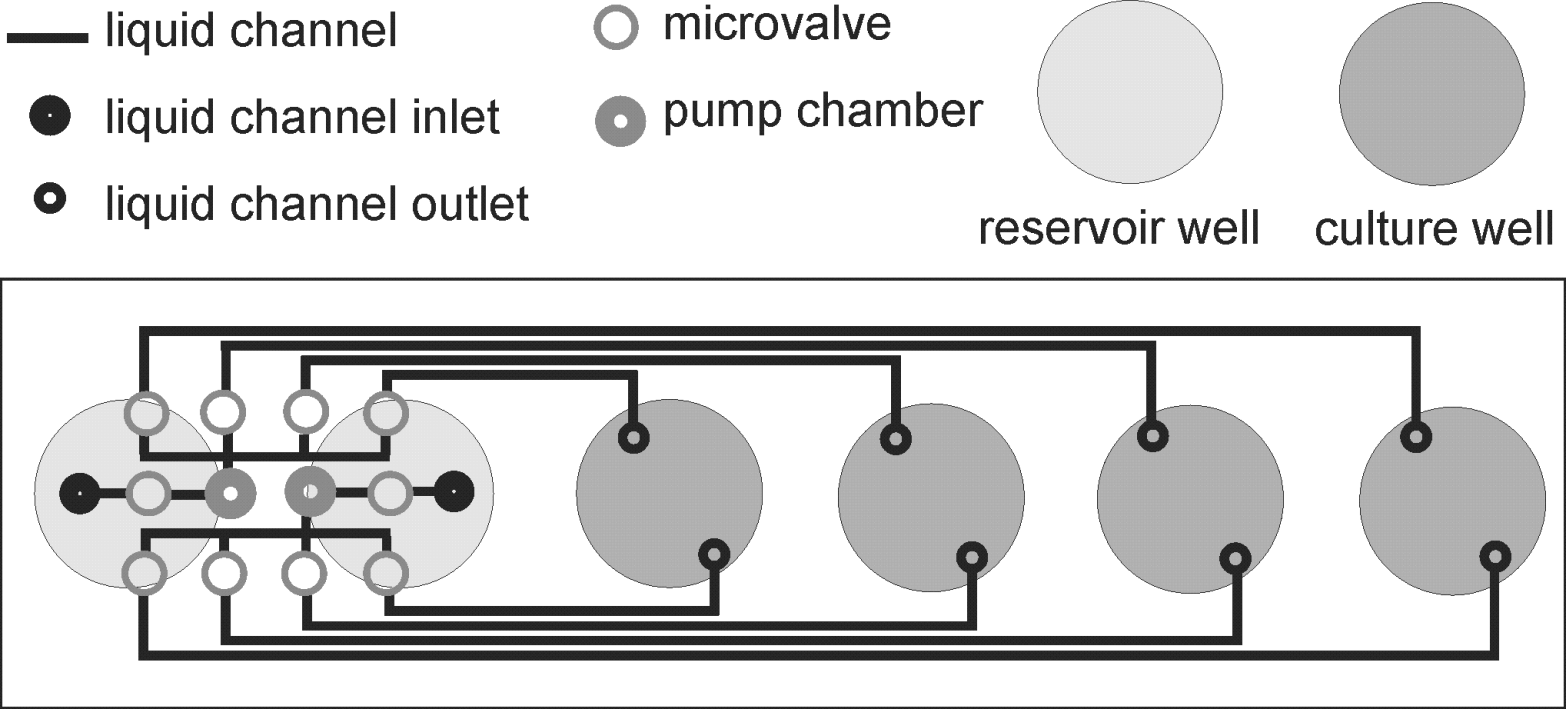
**Alignment of channels, microvalves and pump chamber in microfluidic chips**.

Figure 2 presents a cross-section scheme of the microfluidic MTP. The applied prototypes are composed of a Flowerplate body (m2p-labs GmbH, Aachen, Germany) in which the bottom is replaced by a polystyrene microfluidic chip. The chip is made by injection molding and is as large as a microscopic object slide (75 mm × 25 mm). Glued to the Flowerplate body, one chip covers 6 wells in a row of the plate. Therefore, 2 reservoir wells and 4 culture wells can be addressed with this prototype chip (Figure 3).

The microfluidic chip itself harbors the fluid channels (125 μm × 250 μm) which connect the reservoirs with the culture wells and are sealed with a polystyrene membrane (thickness 50 μm). Besides sealing the channels, this membrane is essential for the microvalve and pumping functions. The microvalves and the pump are actuated by pneumatic pressure (2 bar). The corresponding pneumatic connections are integrated directly in the shaker tray. Since the microvalves and the pump chambers are grouped together on one side of the microfluidic chip directly underneath the reservoir wells (Figure 3), this part of the MTP can be placed on top of the pneumatic connections in the shaker tray without hindering the optical measurement in the culture wells. Moreover, this design allows one to simply clamp the microfluidic MTP on top of the pneumatic connections in the shaker tray and, thus, avoid the manual and elaborate connection of the microfluidic MTP to every single tube to the external pneumatic valves.

Figure 2 illustrates the function of the pH-control and the substrate feeding with the microfluidic MTP. In order to control the pH (Figure 2A-C), the dosing of acid or base is regulated by opening the microvalve in front of the particular culture well. The microvalve consists of a valve cavity and the membrane, which represents the active element of the microvalve (Figure 2A). The microvalve is closed by applying pneumatic pressure (2 bar) to the membrane via the pneumatic connection in the shaker tray (Figure 2B). Hence, the membrane is pressed upwards into the valve cavity and thereby closes it. The microvalve is accordingly opened by applying pneumatic pressure from above to the liquid in the reservoir (0.1 bar) and releasing the pressure from the pneumatic connection in the shaker tray. Hence, the reservoir liquid is forced into the channel of the microfluidic chip and pushes the membrane down, thereby allowing the liquid to pass through (Figure 2C). Since the pH-value is measured inside each culture well by means of optodes, a direct feedback control can be established which changes the valve opening times. The duration of these opening times is calculated by a LabVIEW program based on the actual difference between the setpoint and the actual pH-value as well as the differences during the last measurement cycles.

In contrast to the closed-loop control of the pH-value, the substrate dosage is an open-loop control, because the result of the feeding, i.e. the substrate concentration, cannot be measured directly. Therefore, the flow of substrate needs to follow a given feeding profile. This cannot be accomplished just by determining the valve opening times. Not only does the flow through the microchannels depend on the valve opening times but also on the pneumatic reservoir pressure and the channel length. To solve this challenge, one additional microvalve and one pneumatically driven pump chamber have been introduced in the liquid channel between the reservoir and the culture wells (Figure 2D). As depicted in Figure 2E the pump chamber is filled by applying pneumatic pressure from above to the liquid in the reservoir while the exit valve is closed. Afterwards (Figure 2F), the entry valve between pump chamber and reservoir is closed, and the exit valve to the specified culture well is opened. By applying pneumatic pressure to the membrane underneath the pump chamber, the membrane is then pushed into the chamber and forces a certain volume of liquid (~ 300 nL) out to the reaction well (Figure 2F). Since this dosed volume per pump step is known, a precise flow rate can be established by defining the number of pump steps per time unit.

### Characterization of the Pump Chamber Volume

To establish an exact pump rate, the pump chamber volume was determined before the fermentation experiment. Thereby, the reservoir was filled with 1 mL feed solution composed of 500 g/L glucose (Roth; Karlsruhe, Germany) and 50 μM fluorescein (sodium salt, Fluka, Sigma-Aldrich). The culture wells were filled with 500 μL 0.2 M sodium phosphate buffer (pH 7.5) (Roth). During this experiment, a series of distinct number of pumping steps per time unit was executed, and the fluorescence signal (excitation: 420 nm ± 10 nm; emission: cut-off > 15 nm) in the culture wells was recorded. After calibration with fluorescein solution of known concentrations (0 - 10 μM) in the culture wells, the detected fluorescence signal can be recalculated to establish the precise volume per pump step. The applied pneumatic pressure was 2 bar for the pump chamber and 0.1 bar for the reservoir.

### Cultivation Experiments

All cultivations of *E. coli *K12 were performed in a modified minimal medium according to Wilms et al. [28]. The basic solution consists of 20 g/L glucose; 5 g/L (NH_4_)_2_SO_4_; 0.5 g/L NH_4_Cl; 3 g/L K_2_HPO_4_; 2 g/L Na_2_SO_4_; 0.5 g/L MgSO_4_•7H_2_O; 41,85 g/L 3-(N-Morpholino)-propanesulfonic acid (MOPS); 0.01 g/L thiamine hydrochloride; 1 mL/L trace element solution [0.54 g/L ZnSO_4_•7H_2_O; 0.48 g/L CuSO_4_•5H_2_O; 0.3 g/L MnSO_4_•H_2_O; 0.54 g/L CoCl_2_•6H_2_O; 41.76 g/L FeCl_3_•6H_2_O; 1.98 g/L CaCl_2_•2H_2_O; 33.39 g/L Na_2_EDTA (Titriplex III)]. The pH-value was adjusted to 7.3 with NaOH. In order to apply conditions closer to industrial processes and not to simplify the pH-control experiments inadequately, for the pH-control experiments the concentration of MOPS buffer was reduced down to 10.44 g/L (50 mM instead of 200 mM in standard medium). In both culture vessels, i.e. the microfluidic MTP and the 2 L laboratory-scale fermenter, 2 M solution of ammonia and 1 M phosphoric acid were applied as pH-control reagents. The fed-batch cultivations were performed by feeding a solution of 500 g/L glucose and 70 g/L (NH_4_)_2_HPO_4_. All reagents were of analytical grade and purchased from Carl Roth GmbH & Co. KG (Karlsruhe, Germany). With exception of the pH-adjusting agents which could be considered as autosterile, all media solutions were sterilized by autoclaving at 121°C for 25 min.

To create the precultures, 20 ml minimal medium were inoculated from *E. coli *cryocultures and were shaken over night in 250 mL shake flasks at 350 rpm (5 cm shaking diameter) at 37°C. The main cultures in the microfluidic MTP and the laboratory-scale fermenter, respectively, were created by inoculation from these precultures to a final OD_(600 nm) _of 0.1. All cultivations in the microfluidic MTP were carried out in a temperature-controlled room at 37°C. The plates were sealed with a breathable sealing tape (AB-0718; ABgene, United Kingdom). The main culture in the MTP had a volume of 500 μL and was grown at 37°C under shaking conditions of 1000 rpm at 3 mm shaking diameter. The main culture in the stirred tank reactor had a working volume of 1 L and was grown at 37°C, 950 rpm stirrer speed and a gas flow rate of 1 L/min.

The fermenter (BIOSTAT B plus 2 L; Sartorius AG, Göttingen, Germany) was equipped with two Rushton turbines. The pH-value and the DOT were measured with electrodes (Hamilton; Bonaduz, Switzerland). To determine the OTR and the *k_L_a *online, the respective concentration of oxygen and carbon dioxide in the exhaust gas was measured by means of an exhaust gas analyzer (Rosemount NGA 2000, Emerson Process Management, Haan, Germany) (for detailed description refer to section "Scale-Up"). In the fermenter the pH-control was established by using the embedded PI-controller and a peristaltic pump.

Substrate feeding in the stirred tank fermenter was controlled by the feed balance signal. The setpoint for the P-controller, i.e. the mass of feed solution, which should be dosed from the feed start *t_0 _*up to a distinct time *t*, was calculated by integrating the Equation for the feeding rate *F(t) *[g/h]:

(1) $$F(t)=\left(\frac{\mu_{set}}{Y_{X/S}}+m\right)\cdot X_{0}\cdot V_{0}\cdot \frac{\rho_{F}}{c_{F}}\cdot e^{\mu \cdot (t-t_{0})}$$

The applied feeding parameters were: growth rate *μ_set _*= 0.2 1/h; substrate yield coefficient *Y_x/s _*= 0.5 g/g; maintenance coefficient *m *= 0.06 g/g/h; biomass at feed start *X_0 _*= 3 g/L; culture volume at feed start *V_0 _*= 1 L; density of feed solution *ρ_F _*= 1180 g/L; concentration of feed solution *c_F _*= 500 g/L. Equation 1 was also applied to calculate the feeding rate, i.e. the number of pump steps per time, in the microfluidic MTP (*V_0 _*= 0.0005 L).

### Scale up

The *E. coli *fermentations were scaled up from the microfluidic MTP to a 2 L stirred tank reactor (working volume of 1 L) based on a constant oxygen transfer, i.e. a constant volumetric mass transfer coefficient *k_L_a*. The *k_L_a*-values for cultivations in the Flowerplate at a shaking frequency of 1000 rpm and 3 mm shaking diameter were evaluated using a microRAMOS device. The microRAMOS technology applies a working principle as described by Anderlei et al. [29] for the shake flask RAMOS technology. The gas volume above the MTP, formed by an attached hood, is continuously flushed with humidified air. During recurrent measuring phases in which the air flow is stopped, the oxygen concentration and the overall pressure in the gas volume above the MTP both change due to the respiratory activity of the microorganisms. These changes are monitored by the electrochemical sensors of the microRAMOS device, and the OTR (oxygen transfer rate), CTR (carbon dioxide transfer rate) as well as RQ (respiratory quotient) are calculated [28].

The OTR is defined as the product of the volumetric mass transfer coefficient *k_L_a *and the difference of the oxygen concentration at the gas-liquid interface c* and the current oxygen concentration in the liquid *c_L_*, i.e. the driving concentration gradient:

(2) $$OTR=k_{L}a\cdot (c^{*}-c_{L})$$

If the oxygen concentration in the bulk liquid reaches zero, the oxygen transfer rate reaches its maximum capacity (OTR_max_):

(3) $$OTR_{\max}=k_{L}a\cdot c^{*}=k_{L}a\cdot L_{O_{2}}\cdot p_{O_{2}}$$

The maximum concentration of soluble oxygen c* can be calculated as the product of oxygen solubility in the liquid *L_O2 _*and the partial pressure of oxygen in the surrounding gas phase *p_O2_*. Thus, the *k_L_a*-value of the MTP cultivation was determined from Eq. 3 by applying oxygen limited cultivation conditions (20 g/L glucose, 1000 rpm, 3 mm shaking diameter), and measuring the oxygen partial pressure *p_O2 _*as well as the maximum oxygen transfer capacity (OTR_max_) with the MicroRAMOS device. Even though the oxygen solubility in the minimal medium is not stable over fermentation time and difficult to determine exactly, it can be estimated to be an average value of 0.001 mol/L/bar [30,31]. This simplification has only a minor impact on the absolute *k_L_a*-values, but no influence on the scale-up itself, since the same medium (with the same oxygen solubility) is applied in both the microfluidic MTP and the stirred tank reactor. In replications of these experiments, the *k_L_a*-value for a filling volume of 500 μL and a shaking frequency of 1000 rpm could be determined with an accuracy of 5% to a mean of 460 1/h.

In contrast to the MTP, in the laboratory-scale fermenter, the OTR- and the *k_L_a*-value can be determined online by measuring the DOT with an electrode and the molar fraction of oxygen and carbon dioxide in the exhaust gas *y_O2,out _*and *y_CO2,out _*by means of an exhaust gas analyzer. With the predetermined molar fractions of the incoming air *y_O2,in _*and *y_CO2,in _*as well as the volumetric standard gas flow rate *q_in _*= 1 NL/min and the molar gas volume (*V_M _*= 22.414 L/mol at standard conditions), the following equations are solved online:

(4) $$OTR=\frac{q_{in}}{V_{m}}\cdot \left(y_{O_{2},In}-\frac{1-y_{O_{2},In}-y_{CO_{2},In}}{1-y_{O_{2},Out}-y_{CO_{2},Out}}\cdot y_{O_{2},Out}\right)$$

(5) $$k_{L}a=\frac{OTR}{L_{O_{2}}\cdot p_{abs}\cdot (y_{O_{2},Out}-DOT/100\cdot y_{O_{2},in})}$$

Although this static method of *k_L_a *determination is the most accurate one, it causes difficulties by highlighting the unstable nature of the *k_L_a*-value due to changing medium composition during the prolonging fermentation. Therefore, in preliminary test fermentations the late-exponential growth phase has been chosen as a reference phase to adjust the stirrer speed to 950 rpm, what results in a *k_L_a*-value of approx. 460 1/h at these conditions. In all fermentations performed subsequently a constant stirrer speed of 950 rpm has been applied.

## Results and Discussion

**Figure 4:**
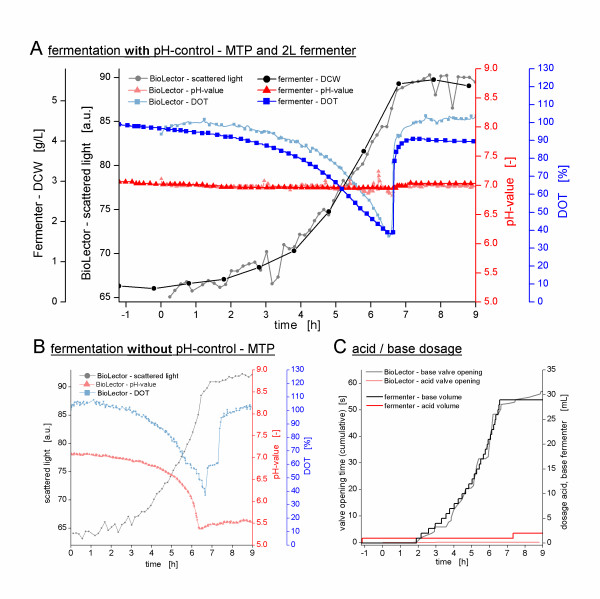
**pH-controlled cultivation of *E. coli *K12 in minimal media supplemented with 10 g/L glucose**. **(A) **Cultivation with pH-control in microfluidic MTP and in 2 L stirred tank reactor (working volume of 1 L) at a matched *k_L_a*-value of 460 1/h. The time-axis shows an offset of 1.2 h due to different duration of the lag-phases in both cultivations performed independently from each other. **(B) **Reference cultivation without pH-control in microfluidic MTP. **(C) **Dosage of 2 M ammonium solution and 1 M phosphoric acid during the pH-control in microfluidic MTP and stirred tank reactor.

**Figure 5:**
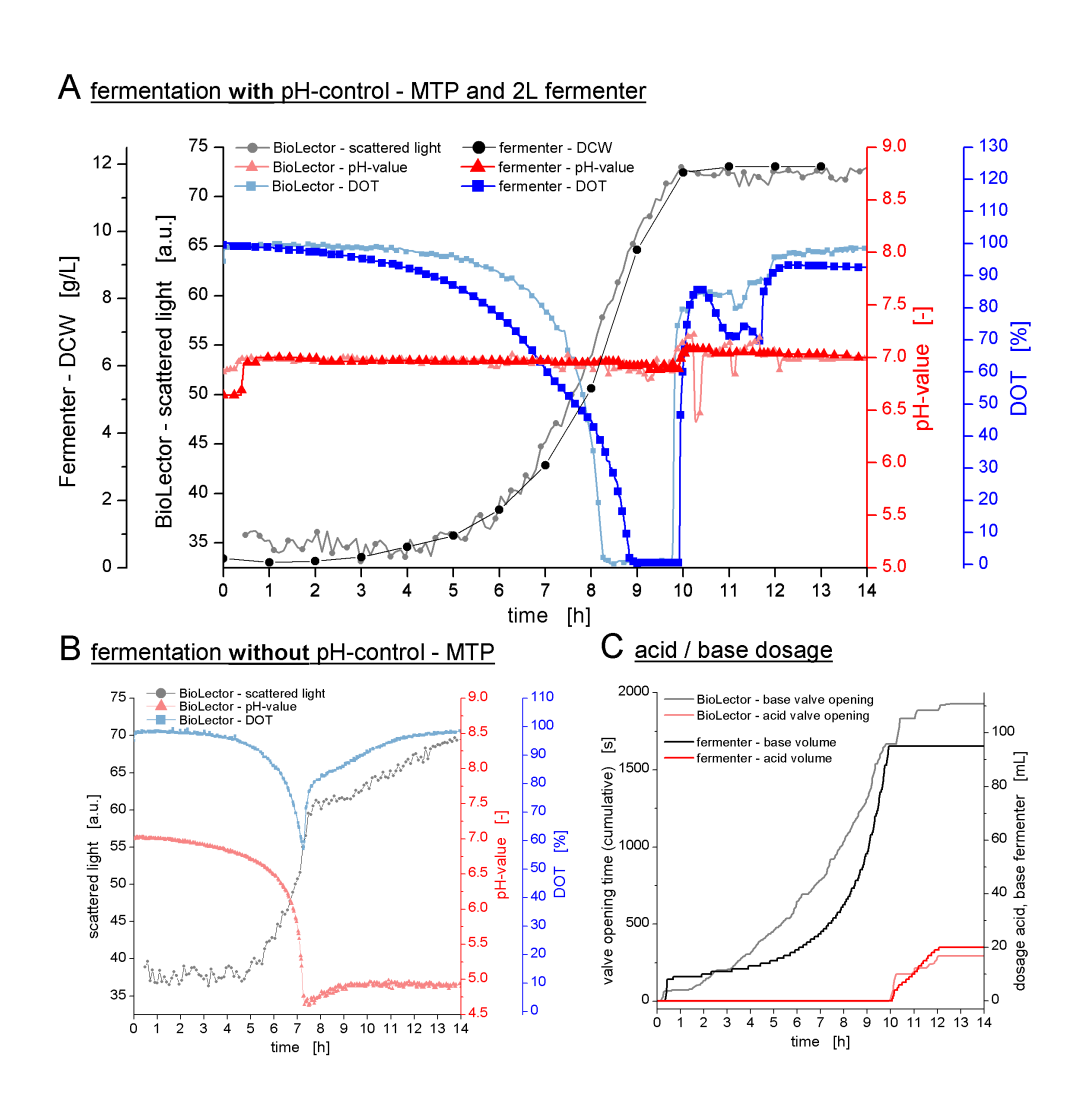
**pH-controlled cultivation of *E. coli *K12 in minimal media supplemented with 30 g/L glucose**. **(A) **Cultivation with pH-control in microfluidic MTP and in 2 L stirred tank reactor (working volume of 1 L) at a matched *k_L_a*-value of 460 1/h. **(B) **Reference cultivation without pH-control in microfluidic MTP. **(C) **Dosage of 2 M ammonium solution and 1 M phosphoric acid during the pH-control in microfluidic MTP and stirred tank reactor.

**Figure 6:**
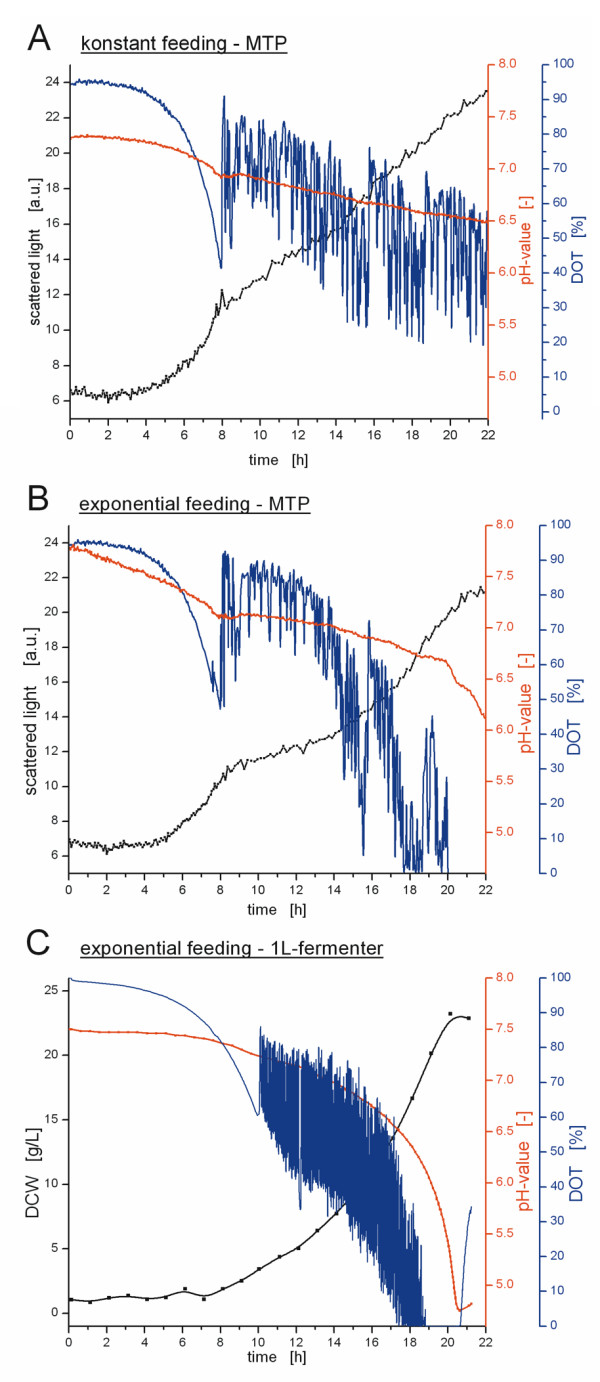
**Fed-batch cultivations of *E. coli *K12 in minimal media initially supplemented with 5 g/L glucose**. The scattered light and DCW are shown in black, the pH-value and the DOT in red and blue, respectively. The feed solution was composed of 500 g/L glucose and 70 g/L (NH_4_)_2_HPO_4_. All cultivations were carried out at a *k_L_a*-value of 460 1/h **(A) **linear feeding in microfluidic MTP with 2 mg/h (= 4 g/L/h with reference to 500 μL initial culture volume) **(B) **exponential feeding in microfluidic MTP with *μ_set _*= 0.2 1/h **(C) **exponential feeding in 2 L stirred tank reactor with *μ_set _*= 0.2 1/h

The newly developed single-use microfluidic MTP and the interaction with its new actuator hardware was tested in pH-controlled batch (Figures 4 and 5) as well as fed-batch fermentations (Figure 6) of *Escherichia coli *K12 in minimal medium. Moreover, the obtained results were compared to cultivations in a 2 L laboratory-scale stirred tank reactor. The performance of the microfluidic process control and its scale-up potential are discussed below.

### Performance of the Microfluidic pH-Control in Batch Fermentation

As Figure 4 shows, by applying a glucose concentration of 10 g/L, only minor differences in the *E. coli *growth behavior can be observed between the pH-controlled (Figure 4A) and the uncontrolled (Figure 4B) fermentations in the microfluidic MTP. The scattered light curve as well as the DOT curve exhibit almost the same courses, whether the pH-value is controlled or not. Obviously, the decrease in the pH-value in the uncontrolled cultivation down to 5.5, caused by the uptake of ammonium from the medium and the production of acidic by-products, has at the most only a minor impact on the *E. coli *culture itself.

However, in the pH-controlled cultivation depicted in Figure 4A, the pH-value is kept in a narrow deadband of 0.03 around the pH-setpoint of 7.0. This is achieved by dosing 2 M ammonia solution to the culture broth. Figure 4C illustrates that this dosage starts together with the growth of the microorganisms and exponentially increases, just like the biomass is exponentially growing.

By applying a glucose concentration of 30 g/L (Figure 5), on the other hand, the effect of the pH-control on the growth of *E. coli *is much more evident. During the first 7 h, the pH-controlled cultivation (Figure 5A) and the uncontrolled cultivation (Figure 5B) showed the same growth behavior. If no pH-control is applied, however, the exponential growth of the culture stops at 7 h (Figure 5B). Here, the scattered light signal stops rising exponentially, and the DOT curve shows a sharp increase. Furthermore, the pH curve is no longer exponentially decreasing after reaching a value of 4.7. This very acidic pH-value indeed inhibits the microbial growth, although there is still glucose in the medium. As indicated by a DOT below 100% and a slightly increasing scattered light signal, after this breakpoint there is still metabolic activity and biomass growth on the remaining glucose, but at a significantly reduced growth rate.

The pH-controlled cultivation with 30 g/L glucose (Figure 5A) shows a significantly different growth behavior. Since the pH-value is actively controlled and, thus, kept in the optimal range over the entire cultivation, the growth is not inhibited by low pH-values. The decrease in the pH due to ammonium uptake and acetate production is compensated by dosing 2 M ammonia solution to the culture broth (Figure 5C). Thus, now no longer inhibited by low pH-values, the microorganisms are able to utilize the complete 30 g/L glucose from the medium. Although, relatively high OTR_max_-value could be reached at the applied shaking conditions (approx. 70 mmol/L/h), a short oxygen limitation between 8 and 10 h could not be avoided. The complete utilization of the 30 g/L glucose results in very high oxygen consumption. As indicated by the sharp increase in the DOT after 10 h, the glucose is depleted and the increase in the scattered light signal stops. Subsequently, the DOT stagnates at around 80% and, for 2 h, 1 M phosphoric acid is dosed to the culture medium (Figure 5C). Both the DOT stagnation and the acid dosing indicate metabolic activity due to the utilization of acidic by-products such as acetate, which has been formed during the first growth phase on glucose.

Compared to the cultivation with 10 g/L glucose, during the cultivation with 30 g/L glucose the suitability of the microfluidic pH-control could be further tested by the successive dosage of both the ammonia solution and the phosphoric acid. As depicted in Figure 4A, the dosing of the ammonia solution resulted in a constant pH-value. However, during the dosing of the phosphoric acid after 10 h, a deviation down to pH-values of 6.4 could be observed, which had to be compensated by subsequently adding ammonia solution to the broth (Figure 4C). Besides reducing the acid concentration and optimizing the controller parameters, this problem will be solved in future by further improving the microfluidic chips, in particular regarding the size of the valve cavity (see also conclusion). Nevertheless, the two pH-controlled fermentations in MTPs described in this paper illustrate that the goal of microfluidic pH-control in MTPs could reproducibly be reached with this technology.

### Scale-Up of the pH-Controlled Cultivations

The direct comparisons of MTP and laboratory-scale fermentations are shown in Figure 4A (10 g/L glucose) and Figure 5A (30 g/L glucose), respectively. The applied scale-up criterion was a constant oxygen transfer on both scales, i.e. a constant volumetric gas transfer coefficient *k_L_a*. By utilizing the microRAMOS device, a *k_L_a*-value of 460 1/h was determined for an *E. coli *cultivation in 500 μL minimal medium in the Flowerplate (1000 rpm, 3 mm shaking diameter) (data not shown). In the stirred tank reactor, the stirrer speed was adjusted to 950 rpm at a constant gas flow rate of 1 L/min to obtain the same *k_L_a*-value as that in the microscale. These conditions were ascertained in previous fermentations. Upon comparing the DOT-curves obtained, the same oxygen transfer was reached in both scales. In both pairs of fermentations, i.e. with 10 g/L and 30 g/L glucose, the DOT-curves in the MTP and in the fermenter, respectively, show similar dynamics. This high parallelism on the DOT kinetics in both scales corroborates the close match of the *k_L_a*-values and the successful scale-up of the fermentations. However, the *k_L_a*-value is naturally changing during the fermentation due to varying broth composition, viscosity, surface tension and oxygen solubility [21]. These changes differently affect the *k_L_a *depending on the aeration method (surface aeration in MTP vs. bubble aeration in the STR) and, therefore, most probably cause the small differences between the DOT-levels observed in Figures 4A and 5A. Moreover, the observed variances might be attributed to differences in the sensitivity of the electrochemical and the optical DOT measurement at different DOT levels. However, these observations may be a target for further investigations. As far as the authors know, this is the first time that a direct comparison of DOT kinetics in such different systems is published.

Besides the oxygen transfer, the courses of the biomass-curves additionally prove that the scale-up was successful. In both pairs of fermentations, the curves of the MTP and the stirred tank reactor can be exactly matched, what reveals that both cultures grow exactly with the same rate. The linear correlation between both biomass measurements - the scattered light and the dry cell weight (DCW) - has been shown in literature [19] and, thus, allows directly comparing the two curves without an elaborate calibration in this distinct experimental setup.

In Figures 4C and 5C, the dosage curves of the pH-control reagents are shown. Also here, a good agreement was observed between the dosing curves in the microfluidic MTP and the fermenter. The differences in the courses of the base dosing in the experiment with 30 g/L glucose (Figure 5C) are caused by a nonlinear correlation between the valve opening time and the dosed volume at very small valve opening times of a few milliseconds (data not shown). At these small valve opening times as they are applied at the start of the fermentation, only a small amount of base can pass the microvalve. As the base dosing and, therefore, the valve opening time increases towards the end of the fermentation a proportionally higher amount of base per valve opening reaches the culture well. In conclusion, the valve opening times are disproportional high at the beginning and disproportional low at the end of the fermentation and result in a more linear course of the base dosing compared to the exponential behavior in the stirred tank reactor.

### Performance of the Microfluidic Substrate Feeding

Figures 6A and 6B depict fed-batch cultivations performed in the microfluidic MTP. Both fermentations start with a batch phase, since the medium has been supplied initially with 5 g/L glucose. After 8 h the exponential growth phase stops, which is clearly visible by the change of the slope in the scattered light, DOT and pH signals. At this time, when the substrate feeding was started manually, the culture continues to grow, however, under glucose limited conditions. This fed-batch-typical glucose limitation is evident from the DOT signal. Since the feeding rate allows only growth at a rate lower than the maximal growth rate, less oxygen is consumed and the DOT can be stabilized above 0%. Another proof that the culture is limited by the substrate feeding is the appearance of recurrent perturbations in the DOT signal. These small interruptions are caused by the pumping steps. Once a pumping step is executed, the microorganisms immediately start to metabolize the dosed glucose and, thus, the DOT decreases. After the dosed glucose is consumed, the DOT rises until the next pumping step occurs thereby causing the observed zig-zag DOT-curve.

Averaging the aforementioned perturbations in the DOT signal and considering only the overall DOT course, one can recognize the particular feeding strategy from the DOT signal. During the cultivation depicted in Figure 6A, a constant feeding rate of glucose of 2 mg/h (= 4 μL/h) was applied, which represents a feeding rate of 4 g/L/h with reference to an initial volume of 500 μL. Consequently, the DOT signal exhibits a linear course. On the other hand, in Figure 6B an exponentially increasing feeding rate for a growth rate of *μ_set _*= 0.2 1/h was set (refer to Eq. 1). Thus, the DOT shows an exponential decrease. Not only does the DOT curve follow the applied feeding rate, but also the curves of the scattered light and the pH-values exhibit a linear or an exponential behavior, respectively. Therefore, it has been proven that the microfluidic substrate feeding satisfactorily works and allows for establishing various feeding profiles in fed-batch cultivations in MTPs. The exact verification of the overall dosed liquid volumes during substrate feeding is complicated by the interfering evaporation of culture broth from the culture well. However, this value can be estimated from the previous calibration experiment and is typically in a range of 40 μL to 60 μL.

### Scale-Up of the Fed-batch Cultivations

Figure 6C illustrates a fed-batch cultivation with an exponentially increasing feeding rate (refer to Eq. 1) (*μ_set _*= 0.2 1/h) of *E. coli *in a 2 L laboratory-scale stirred tank reactor (working volume of 1 L). Here, the batch phase lasts until 10 h. Once the initial amount of 5 g/L glucose was consumed, the culture follows an exponential feeding pattern. The growth behavior during this glucose-limited fed-batch phase agrees well with that observed in the microfluidic MTP (*μ_set _*= 0.2 1/h) (Figure 6B). The curves of scattered light, DOT and pH-values show similar dynamics. Thus, it is possible to reproduce comparable substrate feeding profiles on both scales.

Admittedly, the direct comparison of the fermentations with exponential feeding also shows differences between the MTP and the stirred tank reactor. As an example, the fermentation with exponential feed at *μ_set _*= 0.2 1/h in the stirred tank reactor, which was performed in parallel to the microfluidic MTP, is shown in Figure 6C. Whereas the fed-batch phase (until the culture reaches oxygen limitation) takes approx. 12 h in the MTP, in the stirred tank fermenter this phase lasts only 9 h. Moreover, the calculated growth rate of the culture in the MTP is only 0.13 1/h during exponential feeding. From this value it could be estimated that the feeding rate in the MTP is approx. 30% below the value determined in the preceding calibration experiment. Another observation that supports this theory is the higher average DOT right from the start of the fed-batch in the MTP. Obviously, less substrate is dosed to the culture well and, thus, less oxygen is consumed, compared to the laboratory-scale fermenter.

The observed differences in the feeding rate of the MTP and the laboratory-scale fermentations are likely caused by the insufficient stability of the pump rate in the MTP. Whereas the substrate feeding in the laboratory-scale fermenter is feedback controlled by weighing of the feed solution and, thus, follows exactly the given feeding profile, in the MTP a feedback control cannot be established and the pump rate has to be calibrated before the fed-batch experiment. Although the working principle has successfully been proven by establishing different feeding profiles which are comparable on both scales, the future challenge is to ensure an exact and time-stable pump rate of the microfluidic substrate dosage. For the prototypes of the microfluidic chips utilized so far, the volume per pump step has been determined in a preceding calibration experiment to be in the mean 300 nL with a temporal variation of around 10%. The challenge of more stable and defined pumping and an improved reproducibility of the substrate feeding will be addressed by improving the microfluidic chip in terms of valve size and membrane material (see conclusion).

## Conclusion

A novel microfermentation system based on MTPs, the so-called 'microfluidic BioLector', has been presented in this paper. This technique utilizes microfluidic chips coupled to MTPs to realize pH-controlled batch as well as fed-batch fermentations in culture volumes of several hundred microliters.

The suitability of this microfermentation system for pH-controlled as well as for fed-batch fermentations in MTPs has been proven by fermentations of *E. coli *in minimal medium. Moreover, the scale-up potential of this system has been shown by obtaining equivalent fermentation results compared to a 2 L laboratory-scale stirred tank reactor with a working volume of 1 L. On the base of a similar *k_L_a*-value at both scales, a widely successful scale-up by the factor of 2000 could be reached. Since this system shows high potential for controlled microscale fermentations, a further development of a microfluidic chip which covers the whole MTP is planned. Once that is realized, all 8 rows (each with 2 reservoirs for 4 culture wells) of the 48-well MTP can be addressed, what results in 32 controlled microfermenters per 48-well MTP.

During these investigations, the remaining challenges should be solved by further improving the microvalve design and investigating thinner and more flexible membrane materials. Besides improving the pH-control by reducing concentration of the pH-adjusting agents and optimizing the controller parameters, an improvement of the microvalves would in particular enhance the performance of the microfluidic substrate dosing. Since the currently applied microvalves have a valve cavity volume of approx. 250 nL, a significant volume of liquid is unreproducibly displaced when the valve is pneumatically closed. Smaller microvalves should allow dosing less volume of liquid and thereby improving the liquid dosing. Furthermore, during the currently ongoing fine-tuning of the microfluidic chip, attention is drawn to the applied membrane material. Utilizing a more flexible membrane material should allow an improved reproduction of the membrane deflection not only to the valve cavity but also to the pump chamber and thereby additionally help to guarantee a more reproducible and stably dosed volume in both the pH-control as well as the substrate feed pumping. An alternative route of ensuring a controlled substrate feeding would be the establishment of a feedback control. Besides an online-detectable fluorescent dye in the feeding solution, possible variables for this feedback control could be the scattered light or the DOT value.

The microfermentation system described in this paper combines four advantages: (1) improved microbioreactor design by applying the Flowerplate-MTP, (2) microfluidic process control in disposable MTPs, (3) user-friendly system for connecting the microfluidic MTP to the pneumatic actuator hardware, (4) advanced online-monitoring by the BioLector technology. Integrating the aforementioned properties into one system allows one to use microfluidic MTPs as disposable, ready-to-use cultivation vessels in a user-friendly, "plug-and-cultivate" microfermentation system. In conclusion, this microfluidic BioLector allows user-friendly, cost-effective microscale fermentations that provide high information output and mimic large-scale bioprocesses. This is the mandatory basis for reliable process development and subsequent scale-up.

## Abbreviations

OTR: oxygen transfer rate [mol/L/h]; q_in: _volumetric gas flow rate [NL/L/h]; V_M_: molar gas volume [NL/mol]; y_O2,out_: mole fraction of oxygen in exhaust gas [mol/mol]; y_O2,in_: mole fraction of oxygen in gas inflow [mol/mol]; y_CO2,out_: mole fraction of carbon dioxide in exhaust gas [mol/mol]; y_CO2,in_: mole fraction of carbon dioxide in gas inflow [mol/mol]; k_L_a: volumetric mass transfer coefficient [1/h]; L_O2_: oxygen solubility [mol/L/bar]; p_abs_: absolute pressure [bar]; p_O2_: partial pressure of oxygen [bar]

## Competing interests

The authors declare that they have no competing interests.

## Authors' contributions

MF conceived this biological study and drafted the figures and the manuscript. AB developed the design of the microfluidic chip and the actuator hardware. WM initiated the application of microfluidics for biotechnological systems and supervised the hardware development. SK and LH performed all fermentations, calibrations and data analysis. FK and CM coordinated the project and participated in the development of the microfluidic process control concept. JB initiated the project and supervised the proof of the concept in biological experiments. All authors read and approved the final manuscript.
